# Seroprevalence of leptospiral antibodies in rodents from riverside communities of Santa Fe, Argentina

**DOI:** 10.1371/journal.pntd.0008222

**Published:** 2020-04-24

**Authors:** Tamara Ricardo, Paulina Jacob, Yosena Chiani, María Fernanda Schmeling, Paula Cornejo, Agustina Alejandra Ojeda, Pablo Vicente Teta, Norma Bibiana Vanasco, María Andrea Previtali

**Affiliations:** 1 Consejo Nacional de Investigaciones Científicas y Técnicas (CONICET), Santa Fe, Argentina; 2 Departamento de Ciencias Naturales/Facultad de Humanidades y Ciencias/Universidad Nacional del Litoral, Santa Fe, Argentina; 3 Instituto Nacional de Enfermedades Respiratorias (INER) “Dr. E. Coni”/Administración Nacional de Institutos de Salud (ANLIS “Dr. C.G. Malbran”), Santa Fe, Argentina; 4 Laboratorio de leptospirosis/Facultad de Bioquímica y Ciencias Biológicas/Universidad Nacional del Litoral, Santa Fe, Argentina; 5 Grupo de Investigaciones de la Biodiversidad (GIB)/IADIZA, CCT Mendoza CONICET, Mendoza, Argentina; 6 División Mastozoología/Museo Argentino de Cs. Naturales “Bernardino Rivadavia”, Ciudad Autónoma de Buenos Aires, Argentina; Baylor College of Medicine, UNITED STATES

## Abstract

**Background:**

Leptospirosis is a zoonotic disease that can be transmitted by contact with the urine of infected mammals. Rodents play a mayor role in the transmission of leptospires to humans. The province of Santa Fe reports the greatest number of cases in Argentina. Yet, in this region, there are still knowledge gaps regarding the diversity of rodent species that may be hosts of pathogenic leptospires. The aims of this study were to evaluate the presence of leptospiral antibodies in rodents from three riverside communities of Santa Fe, and to identify factors associated with leptospiral infection.

**Methodology/Principal findings:**

Each community was divided into three environmental settings based on the level of human disturbance, and sampled during two springs (Sep-Oct 2014 and 2015) and one autumn (Mar-Apr 2015). Serum samples of captured sigmodontine and murine rodents were tested for leptospiral antibodies by enzyme-linked immunosorbent assay (ELISA), and microagglutination test (MAT) was used to assess the infecting serovar in seropositive individuals. Factors influencing seropositivity were analyzed using logistic regression models. We caught 119 rodents, of which 101 serums were suitable for analysis. Most frequently trapped species were *Scapteromys aquaticus, Akodon azarae* and *Oligoryzomys* spp., with seroprevalences of 41.3%, 42.9% and 55% respectively. Seropositivity was higher in individuals with an average body condition score and in those that were sexually mature, but in the latter the differences were marginally significant.

**Conclusions/Significance:**

Our results suggest that native rodents may be playing a role in the environmental circulation of pathogenic leptospires and provide relevant information for public health policies in the area.

## Author summary

Rodents are considered as the main reservoirs of pathogenic leptospires, which can cause leptospirosis, a disease that can be severe for both humans and domestic animals. Multiple socio-environmental factors have been found to influence the risk of leptospirosis, and many mammal species can play a role in the transmission of the bacteria. The purpose of this study was to investigate the local conditions and rodent fauna that may influence the risk of leptospirosis in communities located in the floodplains of the Parana river, near Santa Fe, Argentina. We conducted a survey of antibodies against leptospires in rodents from the center, border, and outside of three riverside settlements. We observed a relatively high seroprevalence among captured individuals (42%). Animals with an intermediate body condition were more likely to be seropositive. In most sites, native rodents were dominant over introduced rats and mice and lived close to human dwellings. Given the socio-environmental conditions observed in these settlements and the high frequency of floods that affect them, the presence of potentially infected rodents provide ample opportunities for leptospirosis to affect these communities.

## Introduction

Leptospirosis is a zoonosis of global distribution caused by *Leptospira* spirochetes. More than 250 pathogenic serovars and 40 genomospecies belonging to the pathogenic clade have been identified so far [1–3]. All pathogenic leptospires are capable of infecting multiple animal species. Nevertheless, most serovars tend to be associated with specific maintenance hosts [4, 5]. In reservoir hosts, infection is generally mild or asymptomatic and pathogenic leptospires are chronically maintained in the renal tubules, where they reproduce and are excreted with urine [1, 5]. Humans are incidental hosts, exposure may occur by direct contact with infected animals or indirectly through contact with contaminated water and soil [1, 5].

Rodents serve as maintenance hosts of many zoonotic diseases, being one of the main reservoir species of pathogenic leptospires [1, 4, 5]. Murid rodents of the species *Mus musculus, Rattus norvegicus* and *Rattus rattus* (Muridae: Murinae) are maintenance hosts of the serovars Icterohaemorrhagiae, Copenhageni and Ballum [4, 5]. In recent years, some species of South American sigmodontine rodents (Cricetidae: Sigmodontinae) were also reported as renal carriers of *Leptospira* [6–12]. Introduced and native rodent species usually cohabit and seek food and shelter in the proximity of households, becoming an important source of infection for humans and domestic animals [4, 6]. Some variables related to the biology of these rodents, such as species, sex, sexual maturity or body condition, as well as rodent community composition may affect the ecology of leptospires [4, 9, 13]. Environmental features also can play an important role in the dynamics of leptospires influencing both the behavior of maintenance hosts and the persistence of the pathogen in water and soil [4, 6].

Leptospirosis is a public health problem in Argentina, particularly in slum settlements located in low and flood-prone areas where poor sanitary conditions increase the risk of contact with mud, stagnant water, and infected animals [1, 14–16]. In the country, the most frequently detected serogroups in human cases are Icterohaemorrhagiae, Pomona, Ballum and Canicola [15, 17]. The province of Santa Fe has high incidence rates of leptospirosis, with seasonal peaks during the warm and rainy months, and outbreaks following periods of heavy rainfall or floods [15, 18–20]. Several species of rodents reside in this area, including cavys (*Cavia aperea*), sigmodontines, murines, and the introduced squirrel *Callosciurus erythraeus* (Sciuridae) [21, 22]. Leptospiral infection was reported in Argentina for the four introduced species [7, 14, 23–28] and for the native sigmodontines *Akodon azarae, Holochilus vulpinus, Oligoryzomys flavescens, Oligoryzomys nigripes* and *Scapteromys aquaticus* [7, 10, 23]. In a previous publication, we reported renal carriage of *Leptospira interrogans* in one individual of *S. aquaticus* from Santa Fe [11]. Given that this pathogen is transmitted by a wide diversity of host species, identifying the factors that influence their infection probabilities is key for guiding surveillance efforts that target a broad host range. However, only two studies analyzed factors that could influence over prevalence [7, 23]. In the work of Vanasco et al. (2003) [23], the authors found associations with species and age in rodents from different environmental settings of the city of Santa Fe. On the other hand, Lovera et al. (2017) [7] found associations with abundance and rainfall in rodents from pig farms and dairy farms of the province of Buenos Aires. Nevertheless, small differences in environmental or sanitary conditions within a community may affect the risk of infection in animals and humans [29, 30]. Taking this into account, we hypothesized that higher levels of human disturbance in riverside settlements may increase the probabilities of finding seropositive rodents. The aims of our study were to evaluate the seroprevalence of leptospiral antibodies in native and invasive rodents from riverside settlements of Santa Fe, and to identify risk factors associated with infection considering both environmental variables and rodent characteristics.

## Materials and methods

### Study area

The city of Santa Fe, capital of the province of Santa Fe, is located in northeastern Argentina in the junction of the Paraná and Salado rivers. Santa Fe belongs to the ecoregion of Paraná flooded savanna [31] and has a humid subtropical climate [32]. Annual average temperature is 18-20℃, rainfall ranges from 250 to 400*mm* between spring and autumn and decreases to 60–150*mm* in winter (Servicio Meteorológico Nacional). Interannual variability of precipitation, mainly influenced by the El Niño Southern Oscillation (ENSO), favors the flood of Paraná river in cycles of 2-4 years [33].

**Fig 1:**
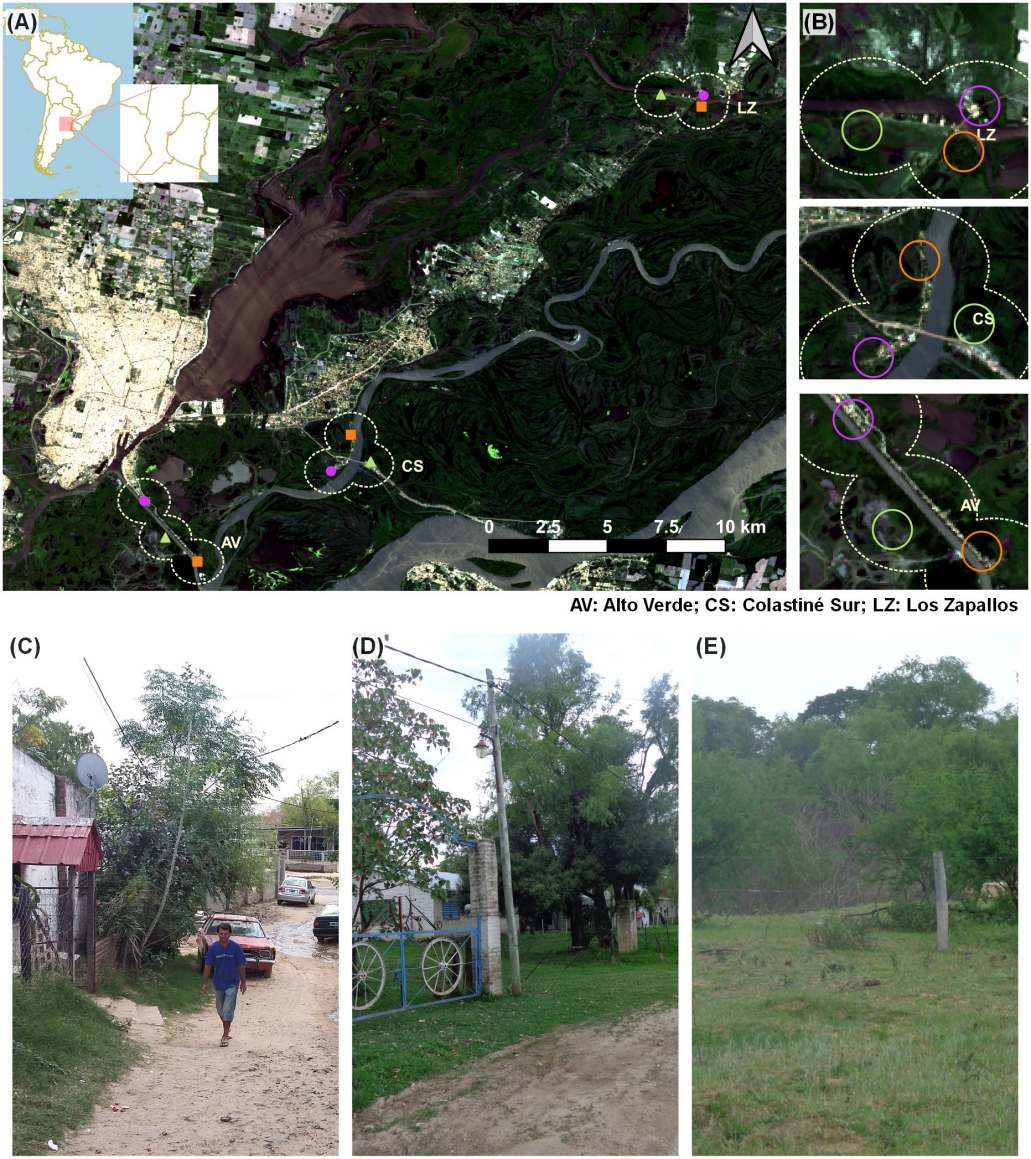
Study sites. (A) Location of the sampling sites within the study area: center sites are depicted in magenta, border sites in orange and natural corridor sites in green. Base map indicates the location of the province of Santa Fe in Argentina and the location of the study area in the province of Santa Fe; (B) Enlarged view of the study sites within the study area; (C-E) Pictures of the study sites: center site of Los Zapallos (C), border site of Colastiné Sur (D), and natural corridor of Colastiné Sur (E). Map created using QGIS 3.0 Girona (QGIS Development Team). Landsat8 OLI/TIRS satellite imagery acquired from U.S. Geological Survey. Vector layers acquired from Natural Earth and Instituto Geográfico Nacional. Photos taken by Dr. Andrea Previtali and Dr. Tamara Ricardo.

We selected the riverside communities of Alto Verde (AV) and Colastiné Sur (CS) located in the outskirts of Santa Fe city, and Los Zapallos (LZ) located 30*km* NE from the city. All the communities are located in the flood valley of the Paraná river, an area of high vulnerability to flooding events and with different levels of deficiencies in infrastructure and services (*i.e*., none of the three communities have sewers or paved streets, and in Colastiné Sur there is also no piped water). More details on the study communities can be found in Ricardo et al. (2018a) [34]. In each community, we established three study sites with different degrees of human disturbance and classified them as center, border, and natural sites depending on whether they were located at the core, the edge or far from the settlement, respectively (Fig 1). Center sites (C) were periurban areas with small dirt roads, open sewers, patches of overgrown vegetation and small dump yards. Border sites (B) were periurban areas of lower human density consisting of a single dirt road with few houses intermixed with natural vegetation. Natural corridor sites (N) were river islands with a single house located in an area consisting of mixed natural habitats mainly used for grazing cattle, pigs, or sheep (Fig 1). Sites from the same community were separated from each other by a body of water and a minimum distance of 1.5 km. Rodent sampling took place between September of 2014 and October of 2015. Daily data on average temperature and rainfall was obtained from the weather stations of Ciudad Universitaria (Centro de Información Meteorológica, Universidad Nacional del Litoral), Alto Verde and San José del Rincón (Municipalidad de la Ciudad de Santa Fe) and from five pluviometers located across the study area (Gobierno de Santa Fe). Data from nearby stations was averaged and assigned to the nearest study community.

### Rodent trapping and sample collection

We conducted three rodent sampling sessions in early spring (Sept-Oct 2014 and 2015) and early autumn (Mar-Apr 2015), coinciding with the periods of reproductive activity of rodents [35]. In each study site, we placed 25 trapping stations separated by 15*m* and consisting of one Sherman Trap and one live-cage trap. In the center sites and some areas of the border sites, traps were placed in the front and backyards of the houses and in vacant lots. Rodents were trapped during three consecutive nights and traps were checked in the morning, replacing the ones with capture success. A more detailed description of sampling protocol can be found in Ricardo et al. (2018b) [11]. We collected blood samples by cardiac puncture and tissue samples by systemic necropsy. For each captured animal we recorded body weight, external measurements, sex, reproductive status, body condition and species. Reproductive status was assessed following the protocol suggested by Herbreteau et al. (2011) [36]. A body condition (BC) scoring technique based on palpation of the hips and lumbar spine was used as a proxy of rodent health status [37]. We identified rodent species in the field and subsequently confirmed the species by inspection of skull morphology at the Museo Argentino de Ciencias Naturales “Bernardino Rivadavia”. If species identification was not possible based on morphological characters, we sent liver and/or lung samples to the molecular laboratory of the Instituto Argentino de Zonas Áridas (IADIZA) for molecular identification through mitochondrial genes [38].

### Serological analyses

Serum samples were analyzed in the Instituto Nacional de Enfermedades Respiratorias (INER). To determine the presence of IgG leptospiral antibodies we used Enzyme linked immunosorbent assay (ELISA) as described by Vanasco et al. (2001) [39]. Sonicated antigen was prepared from cultures of serovar Hardjo, secondary antibodies consisted in a mixture of goat anti-rat IgG (Sigma) and goat anti-hamster IgG (Kirkegaard & Perry Laboratories Inc.). This mixture allows the detection of IgG antibodies of murid and sigmodontine rodents [39]. Samples were tested in duplicate and results were expressed as checked optical density (COD), considering as positive the samples with COD> 2.4 [39]. At this cut-off point, sensitivity and specificity equaled 100% with microagglutination test (MAT) at 1:20 dilution [39]. We did not collect samples from individuals that were found dead in the trap. Additionally, we did not analyzed samples from *C. aperea* and those where the serum was not completely separated from the clot.

Serological typification was performed in ELISA positive animals with more than 50*μl* of serum remaining, using microagglutination test (MAT). Considering the low volumes of serum that can be obtained from the species with small-body size, we used a panel of 10 serogroups (reference strains) recommended by the Argentine Association of Veterinary Diagnostic Laboratories (AAVLD) for detection of animal leptospirosis [40]. This panel is the same that was previously used in the validation of the ELISA test: Castellonis (Castellon 3), Canicola (Hond Utrecht IV), Grippotyphosa (Moskva V), Icterohaemorrhagiae (M20), Pomona (Pomona), Pyrogenes (Salinem), Tarassovi (Perepelicin), Sejroe (Wolfii 3705), Hardjo (Hardjoprajitno) and Hebdomadis (Hebdomadis) [39]. We used the cut-off titre of ≥1:25 to determine exposure to pathogenic leptospires. This cut-off value represents the highest dilution where at least 50% of the leptospires show agglutination [39].

### Data analyses

Data was analyzed with R software [41]. For each site, we calculated species richness (*S*), Shannon diversity index (*H*’) and relative abundance. The composition of rodent community was compared among sites using Chao’s index [42]. Seroprevalence was compared among study sites, sampling sessions, environmental settings and rodent characteristics using either Pearson’s *χ*^2^ or Fisher’s exact tests. Rodent species with small sample sizes (*n* < 10) were excluded from the statistical analysis.

We fitted multivariable logistic regression models to evaluate factors influencing ELISA seropositivity. In order to account for lack of independence among samples from the same site, we fitted mixed-effects models with site as a random intercept. If the variance component of the random intercept was < 1e^-3^, we fitted conditional logistic regression models with study site as stratum. In order to handle infrequent factor levels, similar levels were combined into the same category. Taking into account that IgG antibodies do not develop immediately after infection, we set a lag of two weeks prior to capture date as the minimum time since infection and constructed a set of weather variables for temperature (℃) and rainfall (*mm*). These variables included cumulative rainfall, averaged mean temperatures, the minimum of minimum temperatures and the maximum of maximum temperatures in the previous 30, 60 and 90 days. We compared the fit of multivariable models that included rodent variables and either a weather variable or sampling session using second-order Akaike Information Criteria (AICc). Non significant explanatory variables were removed from the candidate model with the lowest AICc using manual backwards selection. Coefficients of the final model were presented as Odds-ratio (OR) and their 95% confidence interval (95% CI). The level of statistical significance was set at *α* = 0.05.

### Ethics statement

Approval to conduct the study was obtained from the Ethics Committee of the College of Veterinary Sciences of the Universidad Nacional del Litoral (Protocol Number: 14681). Permits for conducting rodent sampling were granted by the Secretary of Environment of the Province of Santa Fe (Protocol: 02101-0013436-1, Resolution: N° 285, Nov. 12, 2013). Trapping and euthanasia of animals were conducted following the recommendations of the American Society of Mammalogists [43]. No protected species were sampled and all efforts were made to minimize animal suffering.

## Results

### Rodent community

We caught 119 rodents of six native species (*A. azarae, C. aperea, Holochilus chacarius, O. flavescens, O. nigripes, S. aquaticus*) and three introduced species (*M. musculus, R. norvegicus, R. rattus*) with a sampling effort of 1661 Sherman trap-nights and 1556 cage trap-nights. The majority of captures (79.8%) corresponded to *S. aquaticus* (45.4%), *A. azarae* (17.6%) and *O. flavescens* (16.8%, S1 Table). Individuals of the genus *Scapteromys* and *Oligoryzomys* were identified to the species level by COI and citB sequence analysis.

Approximately the same number of males (*n* = 59) and females (*n* = 60) were captured. The highest number of captures corresponded to the trapping session of Sep-Oct 2015 (57.1%). The proportion of sexually mature individuals differed significantly between sexes (*P* = 0.023), being higher in males (84.7%) than in females (65%). Among sexually mature females, 14 (35.9%) were pregnant and 78.5% of them were trapped in the spring. Body condition score had a median of 2.5 (*IQR* = 2 − 3), 42% of the animals were under-conditioned (*BC* ≤ 2), 44.5% of the animals had a moderate condition (2 > *BC* ≤ 3) and 14.5% were over-conditioned (*BC* > 3). Neither the numerical nor the categorical body condition differed significantly between sexes (*P* > 0.05).

**Fig 2:**
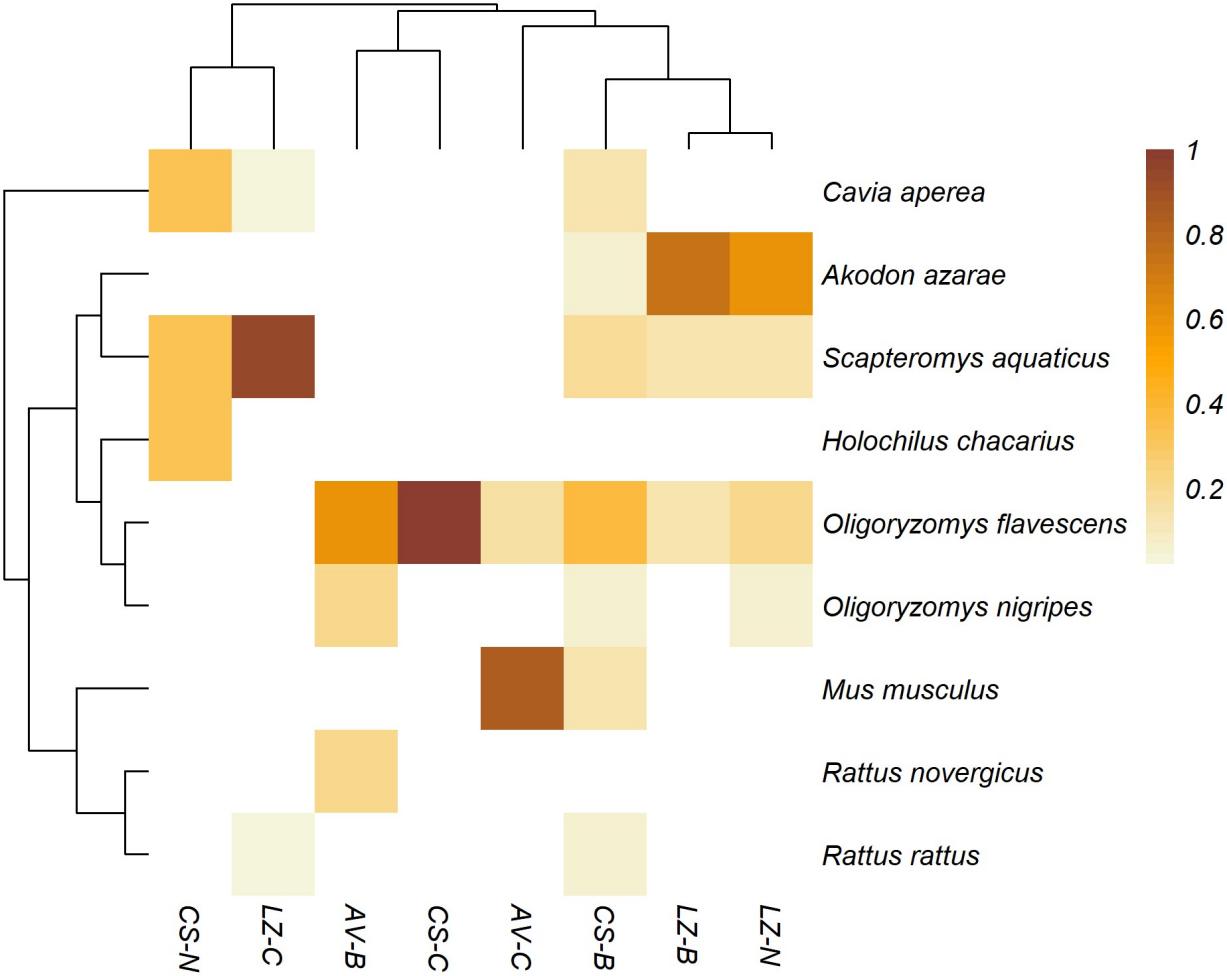
Similarities in the rodent communities between study sites. The top dendrogram shows the grouping of study sites according to Chao’s index. The left dendrogram displays phylogenetic relationships among captured rodent species. Relative abundance of rodents is depicted as a heatmap with darker shades corresponding to higher abundances.

No rodents were caught in site AV-N. Site CS-B had the highest species richness and diversity (*S* = 7, *H*′ = 1.72), while center sites had the lowest. Site LZ-C had the highest rodent abundance (41.2%) but most of the captured individuals belonged to the species *S. aquaticus* (Fig 2, S1 Table). Only one individual of *H. chacarius*, one *R. norvegicus* and two *R. rattus* were captured. The species *C. aperea, S. aquaticus* and *Oligoryzomys* spp. were caught in the three types of environmental settings, while *A. azarae* were not caught in center sites and *M. musculus* were not caught in natural corridor sites (Fig 2, S1 Table). Rodent community composition was more similar among sites LZ-B, LZ-N and CS-B than in sites from the same area or the same environmental setting (Fig 2, S1 Table).

### Seroprevalence of leptospiral antibodies

Of the 119 rodents captured, we analyzed 101 by ELISA, yielding an overall seroprevalence of 41.6% (*n* = 42/101, S2 Table). One of the two captured *R. rattus* was found dead in the trap and could not be tested, the other one as well as the *R. novergicus* and the *H. chacarius* were positive for leptospiral antibodies. The number of seropositive animals was lower in the autumn sampling session, but differences were marginally significant (*P* = 0.082, Table 1). No significant associations were observed between seroprevalence and environmental setting, rodent sex, reproductive status or body condition (Table 1). Seropositivity was associated with study site (*P* = 0.005, S1 Data) and rodent species (Table 1), being negative all of the tested individuals of site AV-C and all of the tested individuals of the species *M. musculus*. After removing the individuals of *M. musculus*, associations with study site and rodent species became non-significant (S1 Data). Serum samples of 18 rodents from the species *S. aquaticus*, *A. azarae*, *O. flavescens*, *H. chacarius*, *R. novergicus* and *R. rattus* were tested by MAT, all of them being non-reactive to the tested panel (S1 Data).

**Table 1:** Seroprevalence (%) of leptospiral IgG antibodies using ELISA by sampling session, environmental setting and rodent variables (n = 101), Santa Fe, Argentina.

Variable	Analyzed	Positive (%)	P
**Sampling session**			0.082
Sep-Oct 2014	29	13 (44.8%)	
Mar-Apr 2015	14	2 (14.3%)	
Sep-Oct 2015	58	27 (46.6%)	
**Environmental setting**			0.264
Border	32	16 (50.0%)	
Center	53	18 (34.0%)	
Natural corridor	16	8 (50.0%)	
**Sex**			0.490
Female	51	19 (37.3%)	
Male	50	23 (46.0%)	
**Reproductive status**			0.375
Immature	25	8 (32.0%)	
Mature	76	34 (44.7%)	
**Body condition** (categoric)			0.180
Under-conditioned	40	14 (35.0%)	
Moderate	47	24 (51.1%)	
Over-conditioned	14	4 (28.6%)	
Species*			0.014
*Mus musculus*	11	0 (0.00%)	
*Akodon azarae*	21	9 (42.9%)	
*Scapteromys aquaticus*	46	19 (41.3%)	
*Oligoryzomys* spp.	20	11 (55.0%)	

* Species with *n* ≥ 10

Logistic regression analysis was carried out for individuals of the species *S. aquaticus, A. azarae*, and *Oligoryzomys* spp and for sites with more than one individual tested. We fitted a set of 14 multivariate models including as explanatory variables rodent species, sex, reproductive status and body condition, as well as one of the constructed weather variables or sampling session. Those models were compared to a base model containing only rodent variables. Given that the variance component of the mixed-effects model were close to zero, we decided to use conditional logistic regression models instead. The multivariate model with the lowest AICc was the base model (Table 2). After removing non-significant variables by a manual backwards procedure, reproductive status and body condition were retained as explanatory variables. In this final model, the odds of being seropositive was associated with a moderate body condition (*OR* = 3.94, 95% CI = 1.27 − 12.20), and marginally associated with sexual maturity (*OR* = 2.90, 95% CI = 0.90 − 9.28).

**Table 2:** Candidate conditional logistic regression models to explain variation in ELISA seropositivity using site as stratum (n = 85).

Model	AICc	Δ*AICc*	K	W
*species + sex + repr. status + BC*	97.5	0.0	6	0.145
*+rain, 60 days*	98.7	1.3	7	0.076
*+min, 60 days*	98.8	1.3	7	0.075
*+min, 90 days*	98.8	1.4	7	0.072
*+rain, 90 days*	98.9	1.4	7	0.071
*+avg, 30 days*	98.9	1.4	7	0.071
*+avg, 60 days*	98.9	1.5	7	0.070
*+avg, 90 days*	98.9	1.5	7	0.070
*+rain, 30 days*	98.9	1.5	7	0.069
*+max, 60 days*	99.0	1.5	7	0.067
*+max, 90 days*	99.0	1.5	7	0.067
*+min, 30 days*	99.1	1.6	7	0.064
*+max, 30 days*	99.2	1.7	7	0.061
*+sampling session*	101.4	3.9	8	0.021

*min*: minimum of the minimum temperature; *max*: maximum of the maximum temperature

*rain*: cumulative rainfall; *avg*: average mean temperature; *BC*: categorical body condition score;

Δ*AICc*: differences in AICc between the candidate model and the best model

K: number of effective parameters; W: Akaike weights

## Discussion

In the present study, we detected three species of introduced rodents (*M. musculus, R. novergicus* and *R. rattus*) and six species of native rodents (*C. aperea, A. azarae, S. aquaticus, H. chacarius, O. flavescens* and *O. nigripes*) living in close proximity to human dwellings. Native rodents were dominant in most of our study sites, except for AV-C which have a more urbanized landscape and was dominated by *M. musculus*. More over, we observed the highest species diversity at a border site, and the three lowest diversity index values were recorded at the 3 center sites. Our results suggest that species diversity and richness may decrease with increasing urbanization, as previously reported by Vanasco et al. (2003) [23] and Cavia et al. (2009) [44].

We detected the presence of leptospiral antibodies by ELISA IgG in *A. azarae, S. aquaticus, O. flavescens, O. nigripes* and *H. chacarius*; species that are widely distributed in riparian habitats of Argentina, Uruguay, Paraguay and Brazil [21, 22]. We found relatively high seroprevalences in the species for which we had a good sample size: *O. flavescens* (55%), *A. azarae* (42.9%) and *S. aquaticus* (41.3%). These results, together with those on our previous report [11] and the findings of Vanasco et al. (2003) [23], Lovera et al. (2017) [7] and Colombo et al. (2018) [10] suggest that pathogenic leptospires are circulating among sigmodontines of central-eastern Argentina. Furthermore, the finding of pathogenic leptospires in the kidney of some of these rodent species suggests that they may be shedding the bacteria to the environment [7, 10–12].

Both rats (*Rattus novergicus* and *R. rattus*) tested positive for IgG antibodies, while none of the 11 *M. musculus* were seropositive. This last result, contrasts with results from a study conducted nearby that found a seroprevalence of 34% in this species [23], and those from studies conducted in the province of Buenos Aires that also found seropositive *M. musculus* [7, 24]. The fact that we found no seropositive *M. musculus*, may be related to low circulation of the bacteria in the patch where they were captured, given that most of the individuals (83.3%) came from the same trap line of site AV-C. It has been previously reported that, except in large urban settings, rodents trapped in households tend to have low infection rates [45, 46], and that prevalence may vary even among different areas of the same neighborhood [30].

In contrast to the high seroprevalences detected by ELISA, the results from the MAT conducted in 18 individuals were all negative. This mismatch may be attributed to ELISA being more sensitive than the MAT [1, 39] or to a low antibody response in wild rodents to leptospiral infection [26, 47]. Due to the small body size of some of these rodent species, we were able to draw a small volume of blood, which limited the number of samples that could be tested by MAT and the serogroup panel. Thus, it may be possible that the infecting serogroup/serovar may not be contemplated in the tested panel [48].

The observed associations between ELISA seroprevalence and rodent species or study sites became non-significant after removing *M. musculus* from the analysis. Previous studies have suggested that observed differences in leptospiral infection among rodent species may be attributed to the habitat preferences and capture rates of each species [46, 49]. On the other hand, we did not find associations between seropositivity and sex or reproductive status, which is in contrast to the results obtained by other studies [13, 23, 45, 46].

We found that individuals with an intermediate body condition had a greater probability of being seropositive for leptospiral antibodies. Our results contrast with those of Himsworth et al. (2013a) [30] and Ayral et al. (2015) [50] which found no significant associations between leptospiral infection and body condition. However, Himsworth et al. (2013a) [30] found significant associations with the volume of internal fat, and Vein et al. (2014) [51] found high seroprevalences in coypus with a good body condition. These results may be indicating that native rodents are able to develop subclinical or chronic infections [26, 51]. The results may also be suggesting that there are behavioral aspects involved in leptospiral transmission, i.e. individuals in a good health status may interact more with others and move further distances for food resources, increasing pathogen exposure [30].

No significant associations were detected between the presence of leptospiral antibodies and weather variables. However, since serological tests determine the presence of antibodies and do not provide precise information on the time of infection [13, 23, 52], it is difficult to identify the timing of the weather event that could have influenced the probability of infection. Lastly, we only sampled two springs and one autumn, thus, it would be necessary to conduct a long-term study expanding over several seasons and weather events in order to detect an effect of weather and seasonality over infection probability.

Beyond the aforementioned limitations, we consider that this study represents a significant contribution to the knowledge of the ecoepidemiology of leptospirosis in Argentina. We detected the presence of leptospiral antibodies in native rodents living in close proximity to households and flooded areas. These riverside communities are vulnerable to frequent flooding and are characterized by precarious houses intermixed with patches of overgrown vegetation and small dump sites. These characteristics are common to many small villages located along the floodplain of the Paraná river. Many of the residents of these communities work in the informal market as subsistence fishermen or farmers and have several domestic animals non vaccinated against leptospirosis [34]. These characteristics, as well as deficiencies in sanitary infrastructure creates a favorable environment for rodent proliferation and may increase the risk of infection with leptospirosis. The high diversity of host species found here, including the diverse community of rodents and the abundant domestic animals, together with the socio-environmental conditions that favor survival and transmission of the bacteria, provide ample opportunities for leptospirosis to take place. This scenario calls for interdisciplinary research teams that could shed light into the role played by the different host species on the environmental transmission of leptospirosis and provide critical information that could guide the development of effective preventive measures.

## Supporting information

S1 Table Rodents captured by sampling site and species. (PDF)

S2 Table Results of ELISA tests by study site and rodent species. (PDF)

S1 Data Ricardo, Tamara; Previtali, Andrea (2020), “Data for Seroprevalence of leptospiral antibodies in rodents from riverside communities of Santa Fe, Argentina”, Mendeley Data, V2, doi: 10.17632/s4c7d7b64s.2. (PDF)
