# Supplementary material for: Seroprevalence of leptospiral antibodies in rodents from riverside communities of Santa Fe, Argentina
Source: PLoS Negl Trop Dis. 2020 Apr 24;14(4):e0008222. doi: 10.1371/journal.pntd.0008222 (PMC7182174; doi:10.1371/journal.pntd.0008222)
Supplement: S1 Table — (PDF) [file pntd.0008222.s001.pdf]

Supplementary Table 1. Rodents captured by sampling site and species.

	AV-B *N=5*	AV-C *N=12*	CS-B *N=16*	CS-C *N=4*	CS-N *N=3*	LZ-B *N=15*	LZ-N *N=15*	LZ-C *N=49*
Species								
Akodon azarae	0	0	1	0	0	11	9	0
Cavia aperea	0	0	2	0	1	0	0	2
Holochilus chacarius	0	0	0	0	1	0	0	0
Mus musculus	0	10	2	0	0	0	0	0
Oligoryzomys flavescens	3	2	6	4*	0	2	3	0
Oligoryzomys nigripes	1	0	1	0	0	0	1	0
Rattus novergicus	1	0	0	0	0	0	0	0
Rattus rattus	0	0	1	0	0	0	0	1*
Scapteromys aquaticus	0	0	3	0	1	2	2	46

*AV: Alto Verde; CS: Colastiné Sur; LZ: Los Zapallos; B: border site; C: center site; N: natural corridor site. * One of the captured animals was found dead in the trap.*
