# Supplementary material for: Seroprevalence of leptospiral antibodies in rodents from riverside communities of Santa Fe, Argentina
Source: PLoS Negl Trop Dis. 2020 Apr 24;14(4):e0008222. doi: 10.1371/journal.pntd.0008222 (PMC7182174; doi:10.1371/journal.pntd.0008222)
Supplement: S2 Table — (PDF) [file pntd.0008222.s002.pdf]

Supplementary Table 2. Results of ELISA tests by study site and rodent species.

	[ALL] *N=119*	NEG *N=59*	POS *N=42*	Not tested *N=18*
Study Site: Species				
AV-B: *Oligoryzomys flavescens*	3	1	2	0
AV-B: *Oligoryzomys nigripes*	1	0	1	0
AV-B: *Rattus novergicus*	1	0	1	0
AV-C: *Mus musculus*	10	9	0	1***
AV-C: *Oligoryzomys flavescens*	2	1	0	1***
CS-B: *Akodon azarae*	1	0	1	0
CS-B: *Cavia aperea*	2	0	0	2**
CS-B: *Mus musculus*	2	2	0	0
CS-B: Oligoryzomys flavescens	6	3	3	0
CS-B: Oligoryzomys nigripes	1	1	0	0
CS-B: Rattus rattus	1	0	1	0
CS-B: *Scapteromys aquaticus*	3	0	2	1***
CS-C: *Oligoryzomys flavescens*	4	0	3	1*
CS-N: *Cavia aperea*	1	0	0	1**
CS-N: *Holochilus chacarius*	1	0	1	0
CS-N: *Scapteromys aquaticus*	1	0	1	0
LZ-B: *Akodon azarae*	11	7	4	0
LZ-B: *Oligoryzomys flavescens*	2	2	0	0
LZ-B: *Scapteromys aquaticus*	2	0	1	1***
LZ-C: *Cavia aperea*	2	0	0	2**
LZ-C: *Rattus rattus*	1	0	0	1*
LZ-C: *Scapteromys aquaticus*	46	25	15	6***
LZ-N: *Akodon azarae*	9	5	4	0
LZ-N: *Oligoryzomys flavescens*	3	1	2	0
LZ-N: *Oligoryzomys nigripes*	1	0	0	1***
LZ-N: *Scapteromys aquaticus*	2	2	0	0

*AV: Alto Verde; CS: Colastiné Sur; LZ: Los Zapallos; B: border site; C: center site; N: natural corridor site. Criteria of exclusion: *animal found dead in the trap, **species not detected by secondary antibodies, *** serum sample not collected or inadequately preserved.*
