# Supplementary material for: Seroprevalence of leptospiral antibodies in rodents from riverside communities of Santa Fe, Argentina
Source: PLoS Negl Trop Dis. 2020 Apr 24;14(4):e0008222. doi: 10.1371/journal.pntd.0008222 (PMC7182174; doi:10.1371/journal.pntd.0008222)
Supplement: S1 Data — (PDF) [file pntd.0008222.s003.pdf]

S1 Data

Datasets and R code used in the data analysis can be found in the following site:

Ricardo, Tamara; Previtali, Andrea, (2020), “Data for Seroprevalence of leptospiral antibodies in rodents from riverside communities of Santa Fe, Argentina”, Mendeley Data, v2 doi: 10.17632/s4c7d7b64s.2
